# A Synoptic Overview of Neurovascular Interactions in the Foot

**DOI:** 10.3389/fendo.2020.00308

**Published:** 2020-05-22

**Authors:** Gayathri Balasubramanian, Prashanth Vas, Nachiappan Chockalingam, Roozbeh Naemi

**Affiliations:** ^1^Centre for Biomechanics and Rehabilitation Technologies, Science Centre, Staffordshire University, Stoke-on-Trent, United Kingdom; ^2^King's College Hospital NHS Foundation Trust, London, United Kingdom; ^3^Faculty of Health Sciences, University of Malta, Msida, Malta

**Keywords:** neurovascular, small fibers, small fiber nerve, microcirculation, diabetic neuropathy, foot

## Abstract

Diabetes is a worldwide public health concern as it is associated with various complications. One of the major complications of diabetes is diabetic foot syndrome that results in catastrophic events such as ulceration and amputation. Therefore, the main four strategies of diabetic foot care involve risk prediction, prevention, and early diagnosis and prompt intervention. The drivers of ulceration are multifactorial, and importantly, include microcirculatory changes in the diabetic skin. Cutaneous microcirculation on the foot is greatly influenced by the small fibers which mediate thermal sensation and pain perception in addition to sympathetic activities such as thermoregulation and vasodilation. The interdependence between the neurovascular elements means with the loss of small fiber functions, the corresponding microcirculatory responses may be compromised. Thus, it can be hypothesized that the impairment of the microcirculation may follow the order of the corresponding small fiber nerve dysfunction or vice versa. In this review, select neurovascular investigations that inform the cutaneous microcirculatory and small fiber nerve function in response to pain, cold, and heat and pressure stimuli are reviewed and discussed in this order of sensory loss: the loss of pain, cold, warmth, touch and deep pressure sensation. We also discuss the neurological and vascular characteristics of each of these neurovascular responses. This review highlights the influence of small fibers on cutaneous microcirculation and the need for prospective studies that can determine the course of microcirculatory impairment over time. This, in turn, may help clarify the exact role of microcirculatory changes in the pathway of ulceration. The insights from this review can be pertinent to understand key microcirculatory disturbances and given that the microcirculatory impairment develops at an early stage, relevant interventions can be implemented to possibly reverse or regress the course of the disease. Therefore, knowledge of the neurovascular interactions aids to map the disease progression for early diagnosis and prevention of adverse complications.

## Diabetes and its Complications: A Growing Concern

Diabetes is a growing public health concern worldwide. Diabetes imposes huge health and economic burden across the nations. Since it is a chronic condition, commonly associated with various complications, the direct and indirect costs of care are high. According to the International Diabetes Federation (IDF), the total health-care spending on diabetes has more than tripled over the period 2003 to 2013 worldwide. Hence, diabetes and its related complications are a persisting problem that demand attention.

There is a spectrum of complications associated with diabetes, with the most common ones being retinopathy, neuropathy, nephropathy, peripheral vascular disease, and foot disease. These complications are the consequences of various glycation related changes that occur after the onset of diabetes over time. These complications significantly decrease the quality of life and may progress to become fatal. Hence, prevention and early diagnosis, treatment and constant care remain the cornerstone for diabetes care.

Diabetic foot ulceration (DFU) is one of the most devastating complications of diabetes. Development of DFU is associated with significant impairment of quality of life, decreased mobility, decreased independence, increased morbidity, and mortality and with impact on health care resources. Moreover, it is estimated that around 85% of non-traumatic amputations are preceded by DFU (1). The literature highlights that the annual population-based incidence of DFUs is around 1.9 to 2.2% (2). There are significant costs associated with DFU—a recent estimate has suggested that the National Health Service in England spends more than £1 billion treating the condition, higher than treatment costs of the lung, prostate and breast cancer combined (3). Development of infection can complicate over 50% of all DFUs which further increases the risk of non-healing. The common sites for ulceration are dorsal or plantar aspects of the toes, plantar metatarsal heads, and heel. In some patients, the ulcers heal with re-epithelialization, which is the restoration of the epithelium in the denuded wound area (2). However, in the absence of healing, there can be infections and adverse outcomes such as amputation. Therefore, early diagnosis and prevention are keys for better management. Effective evidence-based prevention programme with strategies for early detection and control are known to reduce the amputation rate by 50% (4). Thus, knowledge synthesis and understanding the risks associated with a foot ulcer, their interactions and role in the development of an ulcer incident is essential. This can potentially throw light on some of the predictive factors to develop strategies for early interventions.

The key drivers for the development of a DFU are complex and multifactorial (5–7). Rarely does a foot ulcerates due to a single underlying cause, and often, there are several extrinsic and intrinsic risk factors that trigger the diabetic foot to ulcerate (5). Whilst the extrinsic factors include trauma, ill-fitting shoes, walking barefoot, key intrinsic factors include peripheral neuropathy and peripheral arterial disease (5). A combination of two or more of these factors increases the risk of ulceration (5). From previous research, it is well-established that the triad of macrovascular disease, neuropathy and mechanical stress are involved in the pathogenesis of diabetic foot ulceration. In the absence of macrovascular issues and occlusive arterial diseases, a neuropathic foot with palpable pulses may imply microcirculation as a causative factor in the development of an ulcer (8–10). The role of microcirculation in foot complications is evident and well-realized in ulceration and delayed wound healing (2, 6, 8, 11). However, there has been no detailed investigation of its specific role or causal relationship in ulceration. Although the microvascular disease cannot be a standalone cause for a DFU incident, the interaction of microcirculation with the triad and its involvement cannot be denied. Moreover, microcirculatory complications commence at a much earlier stage and microvascular functional changes are predicted to occur even in the prediabetes state and progress with time (12). Hence, identifying the impairment and timely targeted interventions may help reverse some of the changes or delay progression of the disease. Therefore, studies that comprehend neurovascular interactions and underpins the core mechanisms are required. Understanding the underlying pathophysiology associated with the multitude of factors that trigger diabetic foot syndrome is a continuing concern within diabetic foot research. More research focused on the fundamental concepts of neuropathy and vascular diseases can be instrumental in understanding the risks that lead to a DFU incident.

## Overview of Interaction Between Neurological and Vascular Aspects in the Foot

There is evidence that patients with diabetic foot syndrome present with both microcirculatory and neurological disturbances. This neurovascular dysfunction affects the microcirculatory response at the tissue level under conditions of physical stress such as injury or infection, and chemical stress such as contact with heat, cold, chemicals. This may be present even in the absence of major macrocirculatory disturbances and the presence of satisfactory blood flow under normal conditions (5, 6, 10). Whilst both small and large fiber neuropathy is known to play a vital role and is predictive of incident ulceration, the role of microcirculation needs further investigation (6). Therefore, it is easier to explore the unknown through what is known in terms of neurovascular interactions.

This review aims at exploring the role of the microcirculation and the neurovascular interactions by appraising the microcirculatory responses mediated by the small fiber nerves and its significance. This review intends to understand some of the neurovascular interactions, especially in relation to small fibers and microcirculation in the diabetic foot. As mentioned earlier, microvascular functional changes are detectable even in the prediabetes state and progress over time with diabetes (12). With the presence of peripheral diabetic neuropathy, a higher degree of dysfunction is observed (12). Probably, understanding microvascular disease progression and tailored investigation, may aid in the early diagnosis of microcirculatory and accompanied small fiber dysfunctions. Plausibly, this knowledge can be translated to effectively predict diabetic foot complications. Consequently, the practical implications from this review can be valuable for screening, early diagnosis, treatment, and enhancing prognosis by devising management and adapting prevention strategies that can change the paradigm of diabetic foot care in future.

The scope of this review is to identify the microcirculatory responses through functional assessment of small fiber nerves and to discuss cutaneous neurovascular interactions in the foot. Hence, this synoptic overview focuses on key investigations and responses of small fiber functions with relation to microcirculation in the diabetic foot. This is to gain some understanding of the thermoregulatory and biomechanical aspects corresponding to the actions of the thermoreceptors and mechanoreceptors that mediate microcirculation of the foot. Select neurovascular investigations that use stimuli such as pain, cold, heat, and pressure are identified and discussed. Furthermore, the investigation of neurovascular responses must aid to isolate the neurological and vascular components to identify the course of the disease. However, the review boundaries are that conventional tests such as Quantitative Sensory Testing (QST), Quantitative Sudomotor Axon Reflex Testing (QSART), electrochemical skin conductance (SUDOSCAN), iontophoresis, and skin biopsies used to test small fiber nerve functions and microcirculation are not discussed. Therefore, only studies that described small fiber nerve function in relation to microcirculatory responses or vice versa using combined neurovascular testing through brief methods were reviewed. Specific insights generated from the literature are presented and discussed below. Besides, an attempt is made to discuss the neurovascular responses in the order of sensory loss associated with small fiber dysfunction based on their response to local anesthesia, where the fibers of less diameter respond first (13, 14). This is to draw attention to the fact that the corresponding microcirculatory responses may follow the same trend. This may be useful in practice for early diagnosis and monitor disease progression concerning diabetic foot.

## Investigating Neurovascular Aspects

Microvascular dysfunction in diabetes plays a crucial role in the development of diabetic complications. In recent years, functional changes of the microcirculation have gained much attention for their potential role in the development of diabetic complications, especially diabetic foot syndrome. The skin is generally preferred to study microcirculatory functions as it is one of the most accessible organs. The skin microcirculatory bed is rich in capillaries whose functional assessment facilitates understanding of pathophysiological mechanisms that lead to microvascular and small fiber dysfunction. The skin has an intrinsic ability to auto-regulate its blood flow that depends on some of the external or internal factors. Such functions are facilitated by a complex regulatory system that includes local regulation of cutaneous microcirculation involving sensory and autonomic fibers (12). Glabrous skin has highly innervated arteriovenous shunts and plays a major role in thermoregulation (12). In contrast, non-glabrous hairy skin has fewer arteriovenous shunts and is primarily involved in defense and nutrition (12). Additionally, there are various nociceptors in the skin.

Nociceptors are peripherally localized sensory receptor neurons which are sensitive to a noxious stimulus or a prolonged stimulus that eventually becomes noxious (15). Nociceptors detect signals from tissues vulnerable to injuries or from damaged tissue (15). The speed of transmission is directly correlated to the diameter of axons of sensory neurons and whether they are myelinated. Most nociceptors have small diameter unmyelinated axons (C fibers) which support conduction velocities of 0.4–1.4 m/s or A fibers whose axons are myelinated and support conduction velocities of approximately 5–30 m/s (Aδ range) (15, 16). The nociceptors that can be found in the skin, joints and viscera exchange messages respond to a wide range of noxious stimuli (15, 17). Following an incident of injury and inflammation, the nociceptors are sensitized by pro-nociceptive mediators, such as prostaglandins, glutamate, kinins, cytokines, extracellular ATP, protons and other tropic factors (18–20). Also, there are various subcategories of nociceptors that respond based on the site of stimuli application and the type of the stimuli such as chemical, thermal and mechanical (15, 21). Stimulation and activation of the terminal branches of the sympathetic and nociceptor fibers result in axon reflex mediated neurogenic inflammatory reaction, sweating and vasodilation (22).

**Figure 1:**
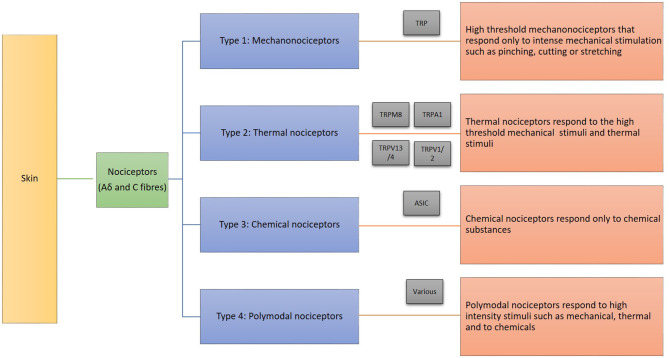
Skin nociceptors and their functions.

The skin nociceptors mediate pain, which can be protective in nature. They differ based on their responses to various types of stimuli. The skin nociceptors are categorized by their function in response to the noxious stimuli as illustrated in Figure 1 (15, 23, 24). Additionally, the skin has polymodal nociceptors respond to high-intensity stimuli such as mechanical, thermal and to chemical substances (23, 24). The skin nociceptors associated with small fibers mediate pain, which can be protective in nature and the skin microcirculation responds to these stimuli. Furthermore, several humoral, neural and external factors are involved in the regulation. In general, cutaneous microcirculatory disturbances in diabetic neuropathy is of interest to understand diabetic foot syndrome and adverse complications such as ulceration and delayed wound healing.

The investigation of the microcirculation in patients with diabetes is an increasing field of interest, fuelled by the availability of novel integrated research techniques used specifically to test microvascular function. Also, these investigations identify the role of small fiber nerves in relation to the microcirculatory responses. This paves the way to understand the neurovascular interaction and contribution to diabetic foot complications. Several non-invasive imaging techniques, mostly laser-Doppler-based methods have been developed in recent times to assess microvascular function in the skin. There are various devices and methods available to evaluate the microvascular changes such as Laser Doppler Flowmetry, Laser Speckle Contrast Image Analysis (LASCA), flow-video microscopy, cannulation measurements of capillary pressure, and transcutaneous oxygen tension measurements. Various provocation tests such as heating, cold, pressure, postural changes and iontophoresis are used to assess the impairment of microcirculation. These provocation tests dependent on the stimulation of the small nerve fibers and are mediated by them to invoke the respective microcirculatory responses. For instance: Endothelium-dependant and independent vasodilation have been studied using the laser Flowmetry through the method of iontophoresis. The indirect effect of the vasoactive substance on skin microcirculation results from the stimulation of C fibers (small fibers), typically through a nerve-axon-related hyperaemic response (25, 26). Thus, the microcirculatory function and the involvement of peripheral nociceptive C fiber function has been assessed simultaneously by measurement of the axon flare-reflex in research. Furthermore, laser-Doppler measurements of the skin microcirculation at the dorsum of the foot following postural change help to understand vascular disturbances in the form of reduced capillary blood flow, observed as an enhanced reduction in skin blood flux, and impaired fluid filtration after sitting up (25). The evidence generated from such studies shows that sympathetic innervation plays a major role in the regulation of skin microcirculation by opening and closing arteriovenous anastomoses and pre-capillary arterioles during postural changes (10, 12, 25). Impairment of endothelium-dependent microvascular regulation is known to correlate closely with the presence of sudomotor dysfunction (12). This microcirculatory impairment is of importance as autonomic neuropathy caused by sympathetic denervation can play a pathogenic role in the development of a diabetic foot; as skin dryness that eventually cracks paves way for infections and ulceration (25). Therefore, neurovascular investigations are useful in understanding and evaluating the association between somatic/autonomic neuropathy and microcirculatory changes.

## Neurovascular Interactions

In polyneuropathy, the small nerve dysfunction is characterized by symptoms such as pain, burning, numbness, and autonomic dysfunction characterized by lack of sweating show a stocking-glove distribution (27). The literature shows that the order of sensation loss is as follows: The loss of pain sensation, cold, warmth, touch and deep pressure upon application of local anesthetics as the smallest fibers respond first (13, 28–30). Research shows an association between microvascular impairment and small fiber neuropathy; microvascular dysfunction contributes to small fiber neuropathy and vice versa as micro-vessels supply the small fibers and small fibers innervate blood vessels (31). Glycation related changes to the microvasculature such as thickening of the basement membrane and altered permeability cause hypoxia of the nerve fibers resulting in the functional loss (32, 33). Increased glucose levels in the cells and tissues stimulate glycolytic and polyol pathways in the peripheral nerve. Furthermore, the modification of proteins with Advanced Glycation End-products (AGEs) and the accumulation of AGEs results in both structural (fiber loss or demyelination of nerve fibers and thickening of the basement membrane of the endothelium in microvessels) and functional damage in the small fiber nerves and microvessels (26, 33). Although glycation related changes in the microvasculature and small fibers are known to occur at a very early stage, it is not clear which precedes the other and how the cycle continues. Based on the severity of the neuropathy and the extent of the small fiber functions lost, the related microcirculatory response may be compromised. If the neurovascular elements are related, with the impairment of small fiber nerve functions, the corresponding microcirculatory responses may be compromised. Thus, the impairment of the microcirculation may follow a similar trend corresponding to small fiber nerve dysfunction (34). This order of sensory loss is upon application of local anesthetics is usually pain, temperature, touch and deep pressure based on the sequential sensory block and for the purpose of this review the neurovascular tests are discussed in this sequence (35). However, the sequence of loss of sensation in case of pathological conditions like diabetes may be different. This depends on the distribution and the number of receptors and of their sensory nerve fibers which are impacted by both aging and diabetes (36). Although the evidence strongly suggests that small fiber neuropathy precedes large fiber neuropathy, there is too little evidence to say which sensory loss mediated by the small fibers is the first one to be lost. The structure and function of small fiber nerves and microvessels are investigated using QST, QSART, electrochemical skin conductance (SUDOSCAN), iontophoresis, and even biopsies (22, 37). Many studies have shown the role of small fiber nerves in microcirculatory responses. Their role is well-established in certain responses more than the other. Previous research has shown the specific role of nerves on certain microcirculatory responses such as following heat provocation tests, topical application of agents such as histamine or capsaicin and iontophoresis (31, 38–42). Whereas, their role in other mechanisms such as Post-Occlusive Reactive Hyperaemia (PORH) and Pressure-induced vasodilation (PIV) needs further exposition. This can be challenging as more than one factors contribute to most of these neurovascular responses. This review focused on providing an account of some of these responses that are non-invasive, less time-consuming and allows for an objective quick assessment of both neuro and vascular function.

## Pain Sensation Mediated By Small Fiber Nerves

Loss of pain or painful neuropathy can be a major cause of foot complications as it can lead to misdiagnosis or late diagnosis of neuropathic complications (43). The pain receptors can be stimulated using certain substances. At the same time, these nociceptors can be sensitized by which the threshold to stimulation is decreased through the use of such substances, which helps to treat the symptoms of pain (44–46).

Sensitization happens following nerve/tissue injury or inflammation through the repetitive exposure to noxious stimuli which triggers an action potential to be propagated to the central terminal via the sensory neurons and the peripheral terminal via the collateral axon branches (17, 47). This instigates the membrane depolarization along with Ca^2+^ influx via the VOCC, inducing the transmitters to be released at the site of the injury and activates the surrounding nociceptors. There are nociception-specific receptors that are present at the afferent terminals: the capsaicin receptor, transient receptor potential cation channel, subfamily V (TRPV1) or vanilloid receptor for capsaicin (VR1) (22, 47). The signaling mechanism pathways involved in the afferent terminal sensitization have included elevation of the Ca^2+^ and activation of G-protein coupled receptors (GPCRs) that results in the elevation of adenylyl cyclase (AC)/cAMP/PKA, phospholipase C (PLC)/inositol triphosphate (IP3)/Ca^2+^ or PLC/DAG/PKC activities. Additionally, neurogenic inflammation can occur through the antidromic release of the transmitters from the collateral branches of the afferent nerves when inflammatory mediators like Substance P, CGRP and neurokinin A are released locally by the afferent neurons (47, 48).

Substances such as histamine, capsaicin, and menthol induce axon reflex or the neurogenic flare response. The most commonly used substance is capsaicin. Capsaicin is a powerful vasodilator and is known to significantly increase skin perfusion (49, 50). Pain-related small fiber functions, symptomology and microcirculatory response are studied through histamine- or capsaicin-evoked axon flare responses are currently visualized by LASCA or photoplethysmography (22, 41, 49). The study by Unal-Cevik (41) characteristics of the flare which depended on the amount of activated small nerve fibers and the function mediated by the C fibers (41). Two components can be isolated from the flare response. The size of the flare and the maximum perfusion represented the neurogenic and vascular components, respectively. The reduction in the size of the flare or its intensity at 5 min following provocation indicated reduced small fiber functions (influenced by the integrity and overlap of C fibers) (41). Besides, the spatial measure, the temporal measures such as prolongation or absence of the latency to reach 3-fold of baseline skin microcirculation denoted diminished small fiber functions (41). On the other hand, the maximum perfusion indicated the microcirculatory function and the spatial distribution of blood pulsation amplitude (BPA) and redness can be attributed to the high perfusion due to vasodilation induced by capsaicin (41, 49). In addition to the monitoring of flare, BPA dynamics measured by imaging photoplethysmography enables to visualize areas highly sensitive to capsaicin, which is indicated to be a novel sensitive non-invasive biomarker of migraine-associated changes in microcirculation (46, 49). However, its role in diabetes is yet to be explored.

It is worth mentioning the influence of certain topical anesthetics on the neurovascular reactions. The use of EMLA cream was found to decrease the responses and pain symptoms, however, it did not completely blocked the small fiber functions (41). Possibly the lower skin innervation layers were only partially inhibited by EMLA cream or certain features of the flare response were not completely influenced by the small fibers (41). Use of lidocaine can induce vasomotor effects similar to capsaicin and also mask its effect, however, perfusion changes do not seem to be influenced by low concentrations of lidocaine (46). These observations raise intriguing questions regarding the nature and extent of small fiber nerve functions on cutaneous microcirculation, especially in the case of neuropathy.

In people with diabetes, peripheral neuropathy causes the loss of these protective responses. The loss of protective sensation and dry skin predisposes the skin to cracks, infection and ulceration. Impaired neurogenic inflammation following capsaicin-induced desensitization can be demonstrated through impaired sudomotor axon reflex and nociceptor axon reflex responses (22). People with diabetes-related small fiber neuropathy present with a range of pain symptoms such as burning sensation, shooting pain, allodynia, and hyperesthesia. In such population, the possible absence of the flare response in people with diabetes may indicate either a severe small fiber dysfunction because of non-receptive superficial C fibers or dysfunction of fibers even in deeper layers of skin. Therefore, further exposition may aid to understand disease progression, small fiber dysfunction and related microcirculatory impairment. This would help to proceed with relevant intervention. For instance, capsaicin is being recommended to treat painful neuropathy and there is ongoing research on whether it will provide additional benefits in terms of improving microcirculation (45, 51). Similar interventions that are tailored and timely may help to reverse impairments and potentially prevent further damage to the neurovascular structures of the skin.

## Heat Perception Mediated By Small Fiber Nerves

Heat stimulus is one of the common provocative tests to study the small fiber functions. During a QST, a range of thermal challenges that are non-nociceptive and nociceptive are used. Heat-induced pain and the threshold is one of the parameters measured which help to assess C fiber functions (37). A most commonly used provocation test used to assess cutaneous microcirculation is local heating as well. This induces nociceptive stimuli-mediated vasodilation and a neurogenic flare by an axon reflex response involving the C fibers. These flare tests are specific to C fibers that can be activated by a stimuli and produces a neurogenic vasodilation (flare response) surrounding the injured site. Apart from thermal stimulus, even electrical stimulus can induce a flare response but such tests are minimally invasive (39, 52). The most commonly used equipment for measuring this effect is the laser Doppler imager (LDI) and the resultant axon reflex, which is a flare response is known as the LDI flare. The dorsum of the feet is preferred for the test as the skin is less influenced by the thermoregulatory blood flow due to the absence of arteriovenous anastomoses (53). The method either involves heating the local area of the skin to 44°C for 20 min or 6 min in a stepwise fashion (44°C for 2 min, 46°C for 1 min and finally 47°C for 3 min) in a temperature-controlled room to evoke the flare followed by scanning the site using an LDI to measure the area (38, 39, 54). The latter protocol is known to produce a significantly larger and consistent response (54, 55). A heating probe that allows direct heating of the skin or probe filled with deionized water is used to assess heat-induced vasodilation and the axon flare response. Similar to the study by Unal-Cevik (41), the LDI flare area or the size of the area with a hyperaemic response is known to be reflective of the small fiber function. Thus, the size of the LDI flare is known to be influenced by the C fiber function, the cutaneous small fiber neural network underneath the probe and its extent (38, 42, 54). The changes in perfusion of the skin immediately beneath the heating probe are a direct response to heating and are reflective of non-neurogenic components involved and may represent the endothelial function (38, 42, 54). Therefore, the intensity of the hyperaemic response depended on the microvascular ability to vasodilate, but on the other hand, the size of the flare was dependent on the small fiber function. This assessment showed reduced neurogenic flare along with microcirculatory dysfunction in people with either type 1 or 2 diabetes (54, 56). Overall, the LDI flare test helps to assess both small fiber nerve dysfunction as well as the associated impairment in cutaneous microcirculation. Further research may aid to isolate the impairment's origin, vascular or neurological, thereby, facilitating to have a better grasp of disease progression.

## Cold Perception Mediated By Small Fiber Nerves

Small fibers and microcirculation are integral for thermal homeostasis in the skin. Although foot temperature is not monitored in routine practice, research suggests that it facilitates risk prediction and early diagnosis of complications (57–60). The common devices used to measure/monitor foot temperature are infrared thermographic cameras, infrared handheld thermometers, Laser Doppler Flowmetry systems and more recently the in-shoe temperature-based sensors designed to fit in prescribed footwear or offloading devices (58).

Plantar thermography is used as a complementary diagnostic method for various foot-related complications (61). Assessing plantar skin temperature can aid to detect the presence of either inflammation or neuropathy (58, 61). To facilitate foot temperature evaluation in people with diabetes, certain provocative tests may be useful (61, 62). One such is the cold stress test, in which the plantar aspect of the feet is covered with thin plastic and immersed in cold water for 1 min or longer (61). Data collection and analysis process involves recording infrared images for baseline and 10 min post-cooling immersion, and calculation of a rewarming index (61).

The cold stress test is also used to study the microcirculation in relation to thermal changes. Through the cold stress test, the afferent nerves that mediate pain and thermal perception in the skin and sympathetic efferent vasoconstrictor aspect are evaluated. The response following the cold stress test might be reflective of a sympathetic vasoconstrictor and the protective vasodilatory activities (63, 64). The initial exposure to cold temperature leads to cutaneous vasoconstriction witnessed by low perfusion (65, 66). However, prolonged exposure to cold increases skin perfusion, a protective hyperaemic vasodilatory mechanism (64, 67). This could be due to the sensitization of the thermal nociceptors to cold. But, the peripheral microcirculatory adaptations to cold exposure-response is known to be unpredictable (67). The cold provocation test is commonly used to study Raynaud's phenomenon, systematic sclerosis, and other conditions (68–71). However, few studies have been conducted to explore the neurovascular responses in people with diabetes (61, 72, 73).

The microcirculatory response to cold stress test is impaired in people with diabetes (74, 75). Increased vascular activity in the digits of people with diabetes (with and without neuropathy), following cold stress, seems to be corresponding with the arteriovenous shunting and the abnormal vascular regulation (74). In accordance with this finding, foot thermography through cold stress test is proven to be beneficial in diagnosing neuropathic complications and mere observation of temperature changes are known to indicate foot at risk (59, 61, 76). Therefore, plantar thermography, which relies on the small fibers function and related vascular responses can be useful in the early diagnosis of diabetic foot complications. There are few imaging techniques such as thermal imaging and Laser Doppler methods used to visualize the skin temperature changes following cold stress tests in people with diabetes to assess the relationship with neurovascular complications in the diabetic foot (49, 61, 74, 76). Studies have demonstrated the changes in skin temperature, perfusion and BPA dynamics in response to thermoregulation following a cold challenge and these changes can be observed in the images using corresponding testing (49, 61, 74, 76). However, there are no studies that identify and isolate the neurogenic and vascular components in the image such as LASCA (49, 61, 76). Such studies may help to further the knowledge of the neurovascular relationship.

## Pressure Sensation Mediated By Small Fiber Nerves

The polymodal mechanothermal receptors in the foot respond to mechanical stimuli such as application of pressure in addition to thermal stimuli. The microcirculatory responses that correspond to the pressure changes are reflected as a change in skin perfusion. The influence of external pressure application on the neurovascular aspects helps to build a foundation that can further be expanded to understand the basics of neurovascular interaction in foot under pressure. The autoregulation of blood flow upon application of extrinsic pressure is known as reactive hyperaemia and the most commonly used provocation tests (Post-occlusive reactive hyperaemia and pressure-induced vasodilation) are discussed in this review.

## Post Occlusive Reactive Hyperaemia (PORH)

Post-occlusive reactive hyperaemia (PORH) is a measure of the reactive hyperaemia to arterial occlusion with pneumatic cuffs. During a PORH test, at occlusion, the blood flow goes to a biological zero followed by a PORH response when the pressure is released. PORH is a transient increase in blood flow because of the induced vasodilation in the organ or tissue following that brief period of the arterial occlusion. During the hyperaemia, the tissue becomes re-oxygenated and reperfusion occurs. PORH is considered to be both endothelial dependant and independent (11, 40). The response is known to be of myogenic, metabolic, neuronal and endothelial (31, 40). Research from as early as in the'90s has demonstrated the involvement of sensory nerves in PORH (13, 77). PORH is mediated by a local reflex involving sensory nerves and an endothelium-derived hyperpolarizing factor, which is known to play a vital role in vasomotor tone for microvessels (40, 78, 79). Although the involvement of a cyclooxygenase product (possibly a vasodilator prostaglandin) was suggested earlier, more recent research shows contradictory results and there is no strong evidence to substantiate the participation of prostaglandins in PORH (13, 40, 78). Larkin and Williams (13) showed that the hyperaemic response could be significantly decreased by the use of topical anesthetic creams through a mechanism that did not alter the vasodilation induced by exogenous calcitonin gene-related peptide (CGRP) or capsaicin (13). The rest flow and blood flow during the recovery seemed to be the same with or without anesthetics. This finding implies that there is a neuronal component involved in the hyperaemic process of the provocation test. The study showed that the use of capsaicin to provoke a microcirculatory response through the stimulation of sensory nerves did increase the blood flow. Similarly, CGRP increased cutaneous blood flow. The increase of blood flow induced by both of these mechanisms were unhindered by the use of topical anesthetics (13). But, the flare produced was abolished (13). This showed that the use of the local anesthetics altered the neurogenic component, which is the axon reflex flare and not the release of endogenic vasodilators (characterized by increased blood flow). The reduced maximum peak flow or magnitude of the PORH response could be attributed to the slower conduction speed of the sensory nerves (80). Sensory nerve function seems to influence the peak perfusion and decrease in time to peak (31, 40). Therefore, in a PORH output, the magnitude and duration of hyperaemia can be considered as the neurogenic component and the increase in blood flow (maximum perfusion/hyperaemia) as the vascular component.

The understanding PORH mechanisms and measures can be valuable as its impairment is found to be associated with both early complications like the presence of peripheral sensory neuropathy in diabetes and late complications such as ulcer (11, 31). PORH is generally measured in the arms but in recent times studies have explored the association of PORH measures at foot in diabetes (11, 13, 40, 81–83). Findings show that for each second increase in time to peak, the likelihood of a participant having a history of foot complication is increased by 2% (11). Therefore, further understanding of the temporal and spatial measures of a PORH response, whether it is indicative of small fiber or microcirculatory dysfunction or both, can help with risk prediction. The neurovascular involvement on the PORH flare was discussed above, however, the isolation of the neurogenic and vascular components for various other PORH measures such as changes in hyperaemic area, hyperaemic repayment, time to recovery, time to latency and such can be of added value. Studies have also highlighted the need to understand this component in order to understand the role of microcirculation and neuropathy which are major contributors to DFU (11, 31). Since microcirculatory and small fiber neuropathy related complications begin at a much earlier stage, such knowledge can help implement appropriate interventions that regress the course of the disease and prevent adverse complications.

## Pressure-Induced Vasodilation (PIV)

Research suggests that Pressure-Induced Vasodilation (PIV) is one of the cutaneous microcirculatory reactive mechanisms to low pressure (84). PIV works through a vascular and neuronal mechanism (84, 85). It results from the interaction of primary afferent nerves and vascular endothelium of skin vessels. The local application of pressure over a particular threshold at a specific location over time may act as a stimulus and the sensations are mediated by the afferent nociceptive C fibers. This response is observed during local application of progressive pressure over time. It is known to be a transient increase in cutaneous blood flow initially before it decreases in response to the stimuli induced by the pressure strain (84–87). This assessment once again shows the relationship between small fiber nerve function and microcirculation. PIV is considered to be more than a transient phenomenon rather an important physiological response allowing the skin to respond adequately to a mechanical stimulus (88). Cutaneous receptors in the skin respond to local mechanical stresses such as local pressure strain (86). These receptors are found to be of mechanothermal nature as the PIV response required certain cutaneous thermal condition (86). The acid-sensing ion channel 3 (Asic3), a neuronal sensor is known to play a pivotal role in causing the vasodilatory response to direct pressure and also for protecting against pressure ulcer (89). The cutaneous Asic3 channels in the skin act as a mechanosensor triggering the microvascular responses through CGRP and produces PIV at low pressures (89). This highlights the fact that small fiber dysfunction as noticed in people with diabetes may result in the absence of certain protective microcirculatory response to temperature or mechanical stimuli or to both. Absence of PIV in people with diabetes showing impaired vasodilation to acetylcholine (endothelium dependent) suggests that PIV is endothelial dependent as well (87). The cutaneous blood flow in response to applied pressure at 5.0 mmHg/min indicated PIV to be absent in the foot of people with type 1 diabetes whereas it existed in healthy subjects (87). This was despite the fact that the study was conducted in a temperature (29.5 +/−0.2°C) controlled environment as low skin temperature in people with diabetes is known to interfere with microcirculation (87). However, a similar study did not observe PIV at 28.7 +/– 0.4°C skin temperature even in healthy subjects and the study suggested the influence of temperature on the mechanism due to the involvement of mechanothermal receptors (86). Further research is required to understand the nature of the mechanoreceptors and their corresponding neurovascular response. Koïtka et al. (87) revealed that in the participants, the non-endothelial-mediated response to sodium nitroprusside was preserved, whereas the endothelial-mediated response to acetylcholine was impaired. This behavior is suggestive of the association between endothelial dysfunction and PIV. On the other hand, the role of small fibers in the response has been implied through the findings of a study that found that vasodilatory axon reflex response to local pressure strain was absent when the capsaicin-sensitive nerve terminals were pre-treated with local anesthetic or chronically applied capsaicin (90).

As mentioned earlier, PIV is a short-lived response, which is believed to be a protective response to non-noxious pressure application. Effect of aging or existing pathology is characterized by the absence of PIV, decreased response or following the PIV the cutaneous blood flow is observed to progressively decrease with the application of increasing local pressure for a prolonged period (86, 91). Upon application of local pressure over time, in comparison to the healthy controls (48.8 mmHg), the cutaneous blood flow was found to decline significantly from baseline at much lower applied pressure (7.5 mmHg) in people with diabetes without neuropathy and with subclinical or clinical neuropathy (6.3 mmHg) (86, 92). The findings suggested that the easily compressible arterial wall and surrounding tissue coupled with impaired response mediated by mechanoreceptors caused an early decrease in cutaneous blood flow (86). Whilst it is apparent that PIV is mediated by C fibers, studies need to isolate the role of the small nerve fibers and the endothelial component possibly through the use of imaging methods.

Largely in people with diabetes, the neurovascular responses to local pressure are compromised and the tissues are very compressible (86, 93). Previous studies have suggested that this microcirculatory response appears to be a protective cutaneous response that relies on the excitation of unmyelinated afferent C nerve fibers (86–88, 94–96). The phenomenon of PIV has been observed even in the pediatric population (96). PIV impairment could contribute to the development of lesions such as pressure ulcers and DFUs (86–88, 94, 95). A recent study demonstrated that the cutaneous vasodilation in response to pressure is decreased in people with DFU in comparison to people without (97). Moreover, these responses are known to be impaired in the cohort of the aging population (84). Thus, the physiological process of aging and progression of pathological conditions of diabetes may worsen the neurovascular functions making certain groups of people (elderly population with diabetes) more vulnerable to complications such as ulcers. Collectively, these findings reveal the significance of protective microcirculatory responses mediated by small fibers that can be important in assessing the risk for ulcers. Therefore, in-depth research analyzing the neurogenic and vascular components and its relationship with local pressure is necessary to understand the tissue vulnerability to ulceration. This can aid with early diagnosis and risk prediction of DFU.

## Conclusion

On the whole, in this review, we have provided an overview of small fiber function and its role in mediating microcirculatory responses. Select tests that are non-invasive and less time-consuming that allows the simultaneous assessment of small fiber functions and the respective microcirculatory response were summarized. The commonly used evaluation methods discussed in this review helps to explore the neurovascular aspects simultaneously in routine practice. Besides, most of these tests facilitate isolating the neurogenic and vascular components of the responses.

In people with neuropathy small fiber dysfunction is known to precede large fiber dysfunction. The small fibers play their role in pain and temperature perception and cutaneous superficial touch/pressure sensations. The impairment of the microcirculation may correspond to the dysfunction of the small fiber nerves. However, prospective studies are required to substantiate it. More research is required to expand the knowledge on these certain responses and their value to understand disease progression. As microcirculatory impairment and small fiber neuropathy are known to precede many other diabetic foot-related complications, outcomes of such research can aid in early diagnosis and better prognosis. Furthermore, whilst the role of microcirculation is well-realized in wound healing, its role played in ulceration remains speculative. Future research in the progression of microcirculatory impairment in the diabetic foot may fetch interesting evidence for resolution.

## Author Contributions

GB and RN made substantial contributions to the conception of the study, data synthesis, and interpretation along with drafting the work. NC and PV contributed to the interpretation of the work and revising it critically for important intellectual content. All authors provided final approval of the version to be published and agreed to be accountable for all aspects of the work in ensuring that questions related to the accuracy or integrity of any part of the work are appropriately investigated and resolved.

## Conflict of Interest

The authors declare that the research was conducted in the absence of any commercial or financial relationships that could be construed as a potential conflict of interest.
